# Functional and Genetic Analyses Unveil the Implication of *hoxa4a* in Zebrafish Craniofacial Development

**DOI:** 10.3390/jdb14020022

**Published:** 2026-05-15

**Authors:** Le Sun, Lu Ping, Fuyu Zhang, Ruzhen Gao, Bo Zhang, Xiaowei Chen

**Affiliations:** 1 Department of Otorhinolaryngology-Head and Neck Surgery, Beijing Tsinghua Changgung Hospital, School of Clinical Medicine, Tsinghua University, Beijing 102218, China; sunledoctor2020@163.com; 2 Department of General Surgery, Peking Union Medical College Hospital, Chinese Academy of Medical Sciences and Peking Union Medical College, Beijing 100730, China; pinglu@pumch.cn; 3 8-Year MD Program, Chinese Academy of Medical Sciences and Peking Union Medical College, Beijing 100730, China; fy_zhang19@student.pumc.edu.cn; 4 Department of Clinical Laboratory, Peking Union Medical College Hospital, Chinese Academy of Medical Sciences and Peking Union Medical College, Beijing 100730, China; 5 Key Laboratory of Cell Proliferation and Differentiation of the Ministry of Education, College of Life Sciences, Peking University, Beijing 100871, China; bzhang@pku.edu.cn; 6 Department of Otorhinolaryngology-Head and Neck Surgery, Peking Union Medical College Hospital, Chinese Academy of Medical Sciences and Peking Union Medical College, Beijing 100730, China; chenxw@pumch.cn

**Keywords:** *hoxa4a*, cranial neural crest cells, craniofacial dysplasia, zebrafish

## Abstract

Microtia–atresia is a rare craniofacial malformation primarily affecting the first and second pharyngeal arches, leading to the deformity of the auricle and atresia of the external ear canal. Its etiology is heterogenous and largely unknown, including both genetic and environmental factors. The *HOXA4* gene has been identified as potentially pathogenetic for microtia–atresia in three twin families. A *hoxa4a* mosaic knockdown zebrafish model was constructed using CRISPR/Cas9. *hoxa4a* was expressed in the mandible during early development in zebrafish, while the F0 mosaic knockdowns exhibited craniofacial malformations with abnormal chondrocyte morphologies. Specifically, *hoxa4a* knockdown reduced cranial neural crest cell proliferation while increasing apoptosis, markedly downregulating chondrogenic markers *sox9a* and *col2a1a*. Consequently, pharyngeal arch chondrocytes exhibited disorganized arrangement and morphological abnormalities, resulting in mandibular hypoplasia. Our findings provide important insights into the role of *hoxa4a* in zebrafish mandibular development and the pathology of microtia–atresia caused by *HOXA4* gene mutations in humans.

## 1. Introduction

Microtia–atresia represents a congenital craniofacial anomaly characterized by underdevelopment or structural abnormalities of the external ear, frequently associated with external auditory canal atresia. Epidemiological studies indicate a prevalence ranging between 0.83 and 5.18 cases per 10,000 live births and a growing trend year on year [1]. This malformation exhibits variable phenotypic expression, presenting as either unilateral (affecting 70–90% of cases) or bilateral (occurring in 10–30% of cases), with potential involvement of middle ear structures. Clinically, microtia–atresia may present as an isolated condition (40–80% of cases) or as a component of broader syndromic manifestations (20–60% of cases) [2]. Multiple signaling pathways work together to regulate neural crest cell development and ear morphogenesis. In mice, knockout of *Wnt5a* impairs the anteroposterior extension of NCC precursors, leading to reduced pinna size [3]. *BMP5*, a gene associated with human congenital microtia, encodes a protein that promotes mesenchymal condensation and osteochondrogenic differentiation [4]. Mutations in *BMP5* cause a short-ear phenotype in mice. The FGF signaling pathway guides NCCs toward ectomesenchymal fates and regulates patterning via *Barx1* and *Dlx2*. This pathway influences NCC proliferation, differentiation, migration, and survival. Mutation of *FGF8* leads to pinna malformation or loss [5]. Furthermore, during pharyngeal arch morphogenesis, retinoic acid signaling interacts with FGF and BMP signaling pathways to coordinate cranial NCC migration and differentiation. Inactivation of the retinoic acid receptor results in hypoplastic arches and cartilage malformation [6]. While certain genes associated with neural crest cell migration and patterning, chromatin modification, fibroblast growth factor receptor signaling, ribosome assembly, and the spliceosome have been found to be involved in monogenic syndromes that result in malformations of the ears or mandible [7], the majority of cases are sporadic and their genetic cause remains unknown.

To identify the genetic causes in patients with sporadic microtia–atresia, we conducted whole-exome sequencing (WES) on six families with monozygotic twins who had different microtia phenotypes [8]. Our analysis revealed recurrent mutations in five genes including a missense mutation (c.920A>C: p.H307P) in *HOXA4*, detected in three families and predicted to be “Damaging” by both Polyphen-2 and SIFT. *HOXA4* (Homeobox A4) is a protein-coding gene, which is located in the A cluster on chromosome 7 and encodes a DNA-binding transcription factor which may regulate gene expression, cell reproduction, differentiation, apoptosis, and signal transduction [9,10]. In humans, the gene has been shown to be associated with diseases including Athabaskan Brainstem Dysgenesis Syndrome (ABDS) [11], Hypospadias [12], colorectal cancer [13], and epithelial ovarian cancer [14].

In zebrafish, the expression patterns of *hoxa4a* within the pharyngeal arches have been studied, revealing its involvement in the anterior–posterior patterning of organs and organ systems during embryonic development [15]. Mutations in *HOXA2* have been reported to result in microtia–atresia [16]. The Hoxa gene cluster plays a predominant role in patterning skeletogenic cranial neural crest cells (CNCCs). Among them, Hoxa2 is uniquely expressed in PA2 CNCCs and serves as a master regulator for second pharyngeal arch identity [17]. Its inactivation in mice causes a homeotic transformation, whereby PA2-derived skeletal elements adopt a PA1-like, Hox-negative fate. Beyond skeletal patterning, Hoxa2 is both necessary and sufficient for external ear morphogenesis. Consequently, hypomorphic or haploinsufficient mutations in Hoxa2/HOXA2 lead to microtia in both mice and humans. Furthermore, *Hoxa2* functions synergistically with Hoxa3 to pattern the derivatives of PA3 and PA4 CNCCs, highlighting its broad and essential role in craniofacial development [18]. Taken together, the above findings suggest that the *HOXA4* (c.920A>C: p.H307P) mutation may acquire pathogenicity by disrupting the DNA binding process and may play an important pathogenic role in the development of microtia–atresia.

In this study, we investigated the potential role of the *HOXA4* (c.920A>C: p.H307P) mutation in the pathogenesis of sporadic microtia–atresia. Using zebrafish as an in vivo model, we characterized the expression pattern of *hoxa4a* during pharyngeal arch development and assessed the functional impact of this patient-derived mutation.

## 2. Materials and Methods

### 2.1. Zebrafish Husbandry and Embryo Preparation

Adult zebrafish were housed under standardized laboratory conditions, featuring a 14 h light/10 h dark photoperiod and a regulated water temperature of 28.5 ± 0.5 °C. Embryonic developmental stages were determined based on established morphological criteria [19]. We obtained the Tuebingen wild-type strain and the Tg (sox10: EGFP) transgenic line (ID: CZ156, ba2Tg/C) from the China Zebrafish Resource Center. To inhibit pigment synthesis where necessary, embryos were exposed to 0.002% 1-phenyl-2-thiourea (PTU; Sigma, Beijing, China, P7629). All experimental procedures involving zebrafish strictly adhered to guidelines approved by the Animal Ethics Committee of Peking University (Protocol No.LSC-ZhangB-3; approved 1 September 2019) and complied with all of the applicable institutional, local, and national regulatory standards.

### 2.2. Bioinformatic Prediction and Molecular Analysis of hoxa4a

Analysis of hoxa4a expression patterns during early zebrafish embryogenesis was conducted using the Spatial Transcript Omics Database (STOmics DB) [20], incorporating both single-cell RNA sequencing (scRNA-seq) and spatial transcriptomic (stereo-seq) datasets. For stereo-seq data interpretation, bin-based annotation methodology was implemented to facilitate spatial gene expression profiling. The tertiary structures of mutant proteins were generated using computational homology modeling via SWISS-MODEL [21], followed by molecular visualization and structural analysis using PyMOL software (v2.5.2). To assess the potential pathogenicity of amino acid substitutions, in silico predictions were performed through two complementary algorithms: SIFT (Sorting Intolerant from Tolerant [22] for estimating mutation impact on protein function, and PolyPhen-2 (Polymorphism Phenotyping v2 [23]) for evaluating structural and functional consequences based on phylogenetic conservation and structural parameters. All web-based tools were accessed during the study period.

### 2.3. Genetic Manipulation of hoxa4a

To achieve targeted gene knockdown in zebrafish, we employed the CRISPR/Cas9 system following a previously reported rapid protocol for generating F0 gene knockouts [24]. Four distinct guide RNA (gRNA) sets (see Supplementary Table S1) were designed and co-injected with Cas9 into embryos at the one-cell stage. The gRNAs were designed using the CHOPCHOP web platform [25] before constructing the Genome-Scale Guide Set. Each gRNA was produced by means of PCR using a forward primer that included a T7 promoter, the target-specific guide sequence, and a scaffold sequence, along with a reverse primer encoding the standard chimeric gRNA scaffold “tracer rev” [26]. Knockdown efficiency was assessed by extracting crude genomic DNA from whole zebrafish embryos, followed by PCR amplification with the indicated primers (Supplementary Table S1) and subsequent sequencing. Because the CRISPR/Cas9 components were injected into one-cell embryos and editing occurs after the first few cleavage divisions, the resulting F0 fish are mosaic for *hoxa4a* mutations rather than uniform germline knockouts.

### 2.4. Whole-Mount In Situ Hybridization

RNA probes were synthesized through in vitro transcription from linearized plasmid templates utilizing appropriate RNA polymerases (Roche Diagnostics, Tokyo, Japan; Stratagene Japan, Tokyo, Japan). The procedures for whole-mount in situ hybridization and immunostaining were conducted following established protocols. Embryonic specimens were initially fixed in 4% paraformaldehyde for 12 h at 4 °C, subsequently dehydrated through a methanol series, and preserved in absolute methanol at −20 °C for long-term storage. The primer sequences employed for gene-specific probe generation comprised *hoxa4a* along with additional primers targeting neural crest markers (crestin, foxd3), craniofacial development genes (*dlx2a*, *barx1*), pharyngeal arch regulators (*tbx1*, *nkx2.3*), and chondrogenic factors (*sox9a*, *col2a1a*), as detailed in Supplementary Table S2. The antisense probe was synthesized with T7 polymerase (Promega, Madison, WI, USA) and a digoxin-NTP mixture (Dig-NTP mix, Roche, Basel, Switzerland).

### 2.5. Cartilage Staining and Immunofluorescence Staining

At 4 dpf, embryos were harvested and fixed through overnight incubation in 4% PFA at 4 °C. After fixation, specimens were rinsed in phosphate-buffered saline supplemented with 0.1% Tween-20 (PBST) and then processed for Alcian blue staining following a published protocol [27]. Additionally, other embryos were subjected to fixation and double staining with wheat germ agglutinin (WGA; Invitrogen) and 4′,6-diamidino-2-phenylindole (DAPI; Sigma-Aldrich, Taufkirchen, Germany).

Transgenic zebrafish embryos expressing EGFP under the sox10 promoter were initially fixed using 4% paraformaldehyde in phosphate buffer. Following fixation, specimens underwent three 20 min washes with phosphate-buffered saline containing 0.1% Tween-20 (PBST). Immunohistochemical analysis was performed using a rabbit polyclonal antibody against phospho-histone H3 (PHH3; dilution 1:400; catalog number sc-374669, Santa Cruz Biotechnology, Dallas, Texas, USA). Apoptotic cells were identified through terminal deoxynucleotidyl transferase-mediated dUTP nick-end labeling (TUNEL) assays, employing the In Situ Cell Death Detection Kit with TMR red fluorescence (product number 12156792910, Roche Diagnostics), following the manufacturer’s protocol. Nuclear counterstaining was achieved with 4′,6-diamidino-2-phenylindole (DAPI), while proliferating cells were detected via PHH3 immunostaining. For specific analysis of cranial neural crest cell populations, quantitative assessments were restricted to cells exhibiting dual positivity for both PHH3/TUNEL markers and EGFP fluorescence, with cell counting performed manually under fluorescence microscopy. All immunofluorescence images were acquired with a Carl Zeiss LSM710 Confocal Microscope (Zeiss, Germany), using consistent settings across all experiments.

### 2.6. Statistical Analysis

For each experimental condition in our phenotypic observations, a minimum of 20 embryos were examined. The findings demonstrated consistent outcomes, with observed effects present in over 85% of the embryos analyzed, and all presented images accurately reflect these results. Quantitative assessments were performed on six randomly selected embryos per experimental group. Statistical significance was determined through unpaired Student’s *t*-tests implemented in GraphPad Prism 6.0 (GraphPad Software, San Diego, CA, USA), with a threshold of *p* < 0.05 denoting statistical significance.

### 2.7. Photography and Image Processing

All brightfield observations were performed using a Stemi 2000-C optical stereomicroscope (Zeiss, Oberkochen, Germany). For ISH, images were acquired with a Stemi 305 stereomicroscope (Zeiss, Jena, Germany) fitted with an AxioCam 208 color camera (Zeiss) and subsequently processed via ZEN 3.1 software. Immunofluorescence images were obtained using a Zeiss LSM 710 NLO laser scanning confocal microscope (Zeiss, Germany) coupled with a Duoscan System. Image analysis and processing were carried out in Zeiss Zen 2009, and final figure assembly was performed in Adobe Photoshop.

## 3. Results

### 3.1. Homology and Expression Profile of HOXA4 in Zebrafish

**Figure 1:**
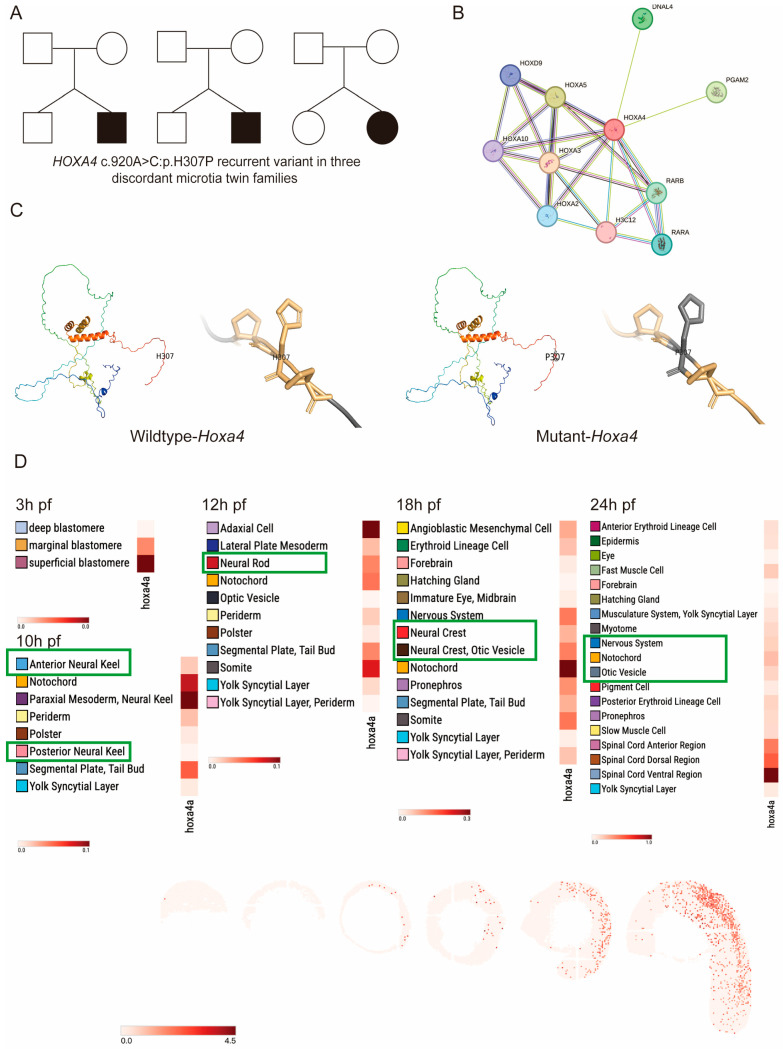
Identification of the zebrafish homologue of *HOXA4* and its related gene. (**A**) *HOXA4* gene schematic in microtia-discordant monozygotic twin families. (**B**) SWISS-MODEL structural prediction indicates dramatic changes in *HOXA4.* (**C**) STRING interaction prediction showed a relationship with the RA pathway. (**D**) Expression profile of zebrafish *hoxa4a*, as determined using the Spatial Transcript Omics Database.

Genetic analysis of three twin pairs affected by microtia revealed a shared *HOXA4* missense mutation (c.920A>C, p.H307P) in all cases (Figure 1A). Bioinformatic analysis also revealed that structural alterations of this gene are functionally associated with the RA pathway and concurrently identified correlations with multiple genes of the *HOXA* family, including *HOXA2*, *HOXA3*, and *HOXA5* (Figure 1B). Computational predictions using both SIFT and PolyPhen-2 algorithms consistently classified this amino acid substitution as deleterious (Figure 1C), providing strong evidence for the potential pathogenicity of this *HOXA4* variant in microtia development. The tertiary structures of mutant proteins were computationally predicted through the online program SWISS-MODEL and subsequently visualized with PyMOL software [8]. Sequence characterization demonstrated that the HOXA4 gene product comprises 320 amino acid residues. Comparative genomic assessment utilizing NCBI BLAST (Version 2.14.0) identified the zebrafish ortholog hoxa4a, which encodes a shorter polypeptide of 245 amino acids. Alignment of the protein sequences between human and zebrafish exhibited 54.7% identity with a highly significant E-value of 7 × 10^−4^, confirming substantial evolutionary conservation between these orthologous proteins.

The temporal and spatial expression profile of *hoxa4a* during early zebrafish embryogenesis was initially explored using the ZESTA database [28]. Analysis of single-cell RNA Sequencing (scRNA) data revealed that, at 10 hpf, *hoxa4a* transcripts were present in both the anterior and posterior regions of the neural keel. At 12 hpf, *hoxa4a* was mainly expressed in the neural rod, whereas at 18 and 24 hpf, *hoxa4a* was expressed in the neural crest and otic vesicle at 18 and 24 hpf (Figure 1D).

**Figure 2:**
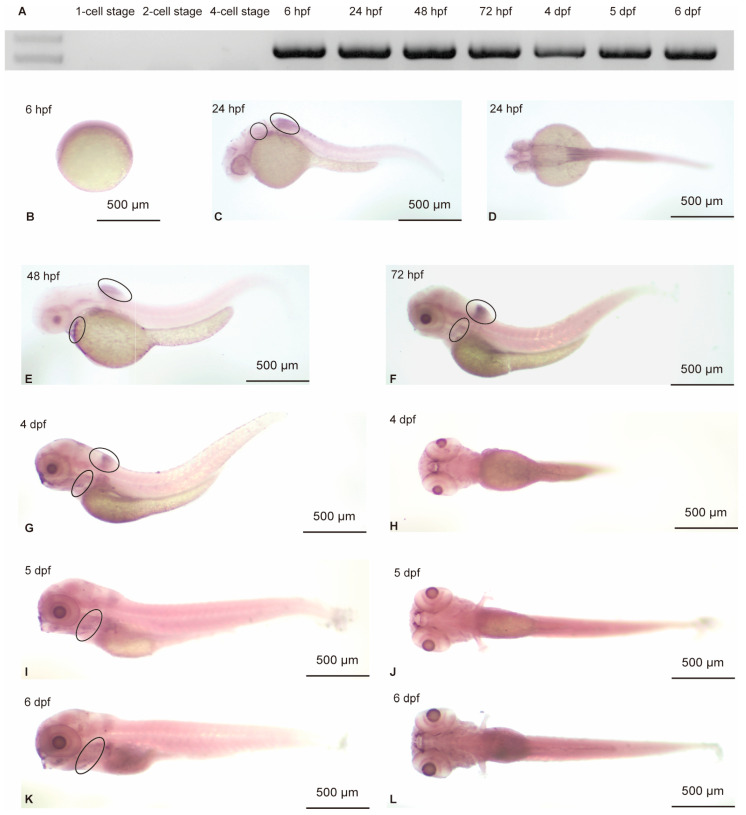
Temporal expression profile of *hoxa4a* during zebrafish development. (**A**) RT-PCR analysis demonstrating that *hoxa4a* transcripts were detectable from 6 hpf through 6 dpf. (**B**–**L**) ISH results confirming the same expression window (6 hpf to 6 dpf) for *hoxa4a*. (**C**,**D**) *hoxa4a* expression in the otic vesicle and dorsal hindbrain at 24 hpf. (**E**–**L**) *hoxa4a* expression in the pharyngeal arches from 48 hpf to 6 dpf.

To evaluate the zebrafish expression profile of *hoxa4a*, RNA was extracted from embryos at different developmental stages for RT-PCR. The expression of *hoxa4a* could be detected beginning at 6 hpf up to 6 dpf (Figure 2A). Whole-mount in situ hybridization analysis conducted on embryos throughout the initial 6 days of development validated these observations (Figure 2B–L), with results consistent with data from the ZESTA database. At 24 hpf, *hoxa4a* transcripts were localized to the otic vesicle and dorsal hindbrain regions (Figure 2C,D). By 48 hpf, expression was evident in the pharyngeal arches, and this expression pattern was maintained through 6 dpf (Figure 2E–L), suggesting a functional role for *hoxa4a* in pharyngeal arch development.

### 3.2. Severe Pharyngeal Malformations Are Induced upon Knockdown of hoxa4a

**Figure 3:**
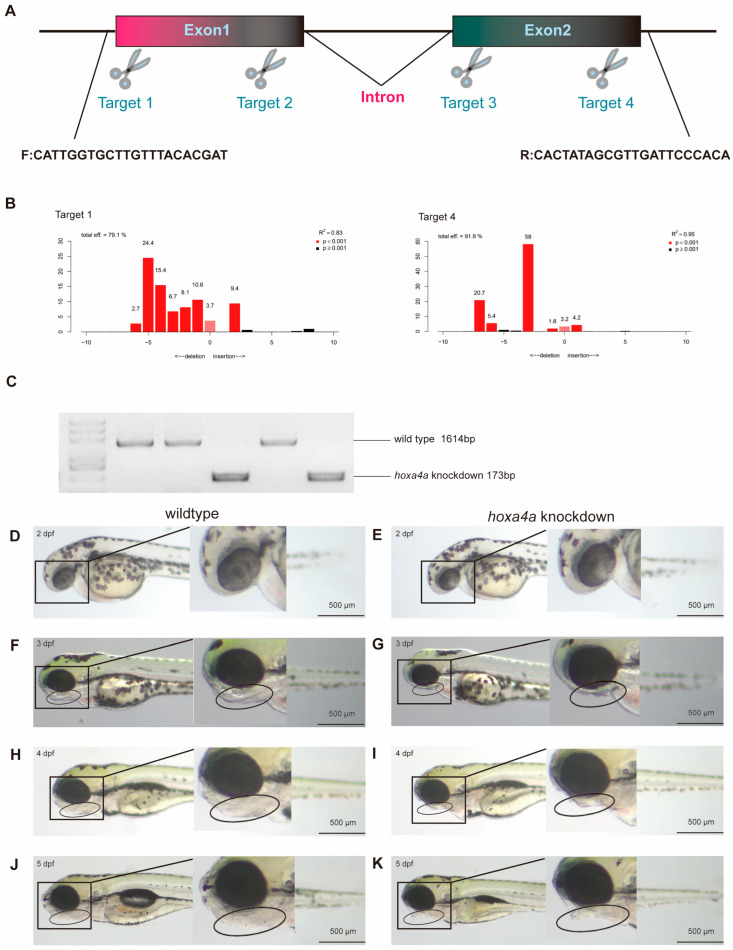
Phenotypic consequences of hoxa4a knockdown. (**A**) Locations of four gRNA target sites on *hoxa4a*, designed for CRISPR/Cas9-based knockdown. (**B**) Representative knockdown efficiencies of individual gRNA targets, as determined by means of PCR. (**C**) PCR evidence showing a large deletion fragment within the mutated alleles. (**D**–**K**) Side-by-side brightfield views of wild-type vs. *hoxa4a* knockdown embryos.

CRISPR/Cas9 [24] was employed to knock down hoxa4a expression, aiming to assess the impact of hoxa4a variants on pharyngeal development. The *hoxa4a* gene consists of two exons, and we have developed two gRNA targets for each exon (Figure 3A, Supplementary Table S1), with each co-injected with Cas9 into one-cell embryos. At 1 dpf, gRNAs for Target 1 and 4 showed knockdown efficacies of 79.1% and 91.9%, respectively (Figure 3B). Additionally, we used Target 1 forward primer and Target 4 reverse primers of the knockout fragment to assess the efficiency of gene knockdown. The sequence between the two primers is 1614 bp, and after a large fragment deletion, only about 173 bp remain (Figure 3C). This allows for clear detection of the mutated bands.

To establish a zebrafish line with *hoxa4a* knockdown, we selected the deletion fragment that exhibited the strongest suppressive effect on *hoxa4a* expression. Figure 3D–K compares the phenotypes of wild-type animals and those with mosaic *hoxa4a* knockdown. During early embryogenesis, individuals lacking *hoxa4a* function appeared morphologically normal, showing no clear defects (Figure 3D,E). Notably, by 3 days post-fertilization (dpf), while control embryos exhibited normal mandibular formation, the *hoxa4a* morphants displayed a complete absence of lower jaw structural development (Figure 3F,G). By 4 dpf, wild-type embryos exhibited clear signs of bladder inflation and continued forward protrusion of the lower jaw, while *hoxa4a* knockdown embryos lacked any such morphological changes in either the bladder or the jaw (Figure 3H,I). At this stage, the divergence between the two groups was strikingly greater than at earlier time points. These morphological abnormalities persisted through 5 dpf, ultimately proving lethal due to respiratory and/or deglutition impairment (Figure 3J,K). Notably, comparative analysis revealed no significant differences in otocyst and otolith development between experimental groups, suggesting that the observed craniofacial and visceral defects represent tissue-specific developmental anomalies rather than generalized developmental retardation.

### 3.3. Knockdown of hoxa4a Results in Pharyngeal Arch Cartilage Dysplasia and Chondrocyte Disorganization

**Figure 4:**
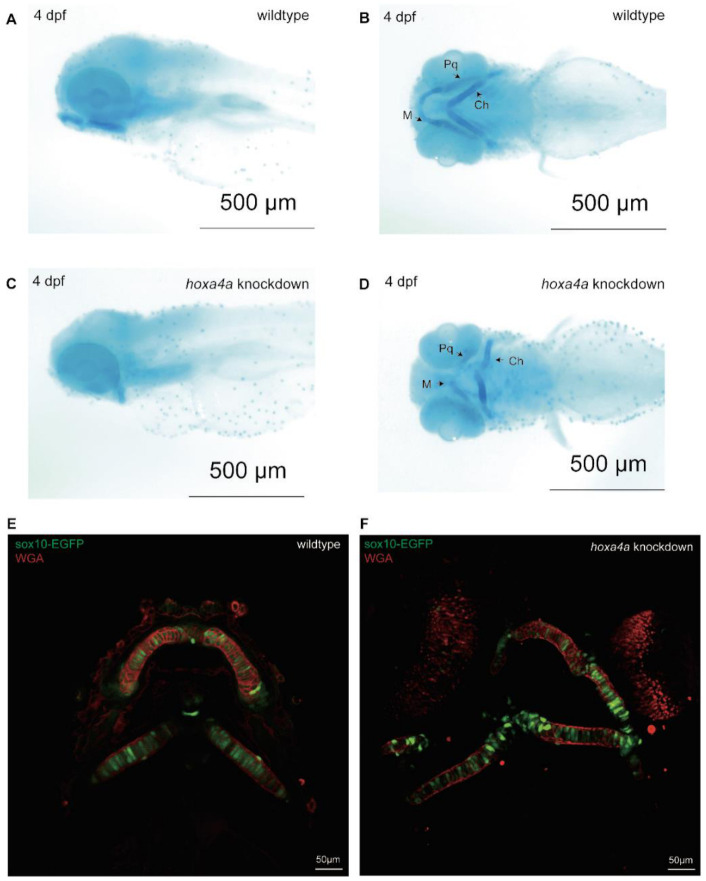
Phenotypic effects of *hoxa4a* knockdown demonstrated through Alcian blue/WGA staining. (**A**,**B**) Wild-type larvae stained with Alcian blue display regular pharyngeal cartilages. (**C**,**D**) By contrast, *hoxa4a* knockdown results in misshapen Meckel’s and palatoquadrate elements. (**E**) WGA reveals well-ordered, morphologically normal chondrocytes in controls. (**F**) Knockdown embryos instead show distorted cartilages and irregular chondrocyte packing.

Comparative analysis of mandibular morphogenesis in wild-type vs. *hoxa4a* knockdown embryos was performed through Alcian blue staining of pharyngeal arch cartilaginous structures at 4 days post-fertilization (Figure 4A–D). The *hoxa4a* knockdown group exhibited significant morphological abnormalities, including incomplete formation and structural deformities of both Meckel’s cartilage and palatoquadrate elements. For more detailed cellular characterization, pharyngeal chondrocytes were additionally labeled with wheat germ agglutinin (WGA) conjugate. Microscopic evaluation revealed pronounced cellular pathology in *hoxa4a* F0 mosaic knockdowns, with Meckel’s cartilage chondrocytes displaying abnormal swelling and disrupted spatial organization (Figure 4E,F), consistent with profound impairment of cartilage differentiation and patterning.

### 3.4. Knockdown of hoxa4a Affects NCC Proliferation and Apoptosis

**Figure 5:**
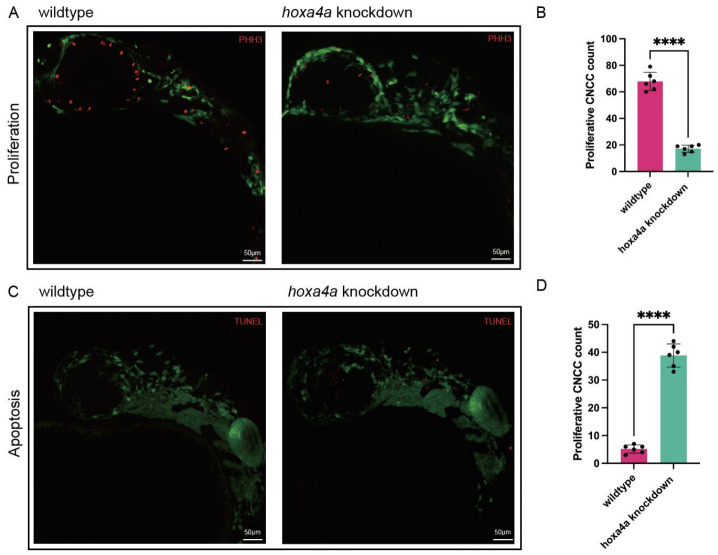
Cranial neural crest cell (CNCC) dynamics in zebrafish embryos. (**A**) PHH3 staining showing proliferation of CNCCs in wild-type and *hoxa4a* knockdown embryos. (**B**) Comparative CNCC proliferation analysis: wild-type vs. *hoxa4a* knockdown (*n* = 6 each). (**C**) TUNEL staining showing apoptosis of CNCCs in wild-type and *hoxa4a* knockdown embryos. (**D**) Comparative CNCC apoptosis analysis: wild-type vs. *hoxa4a* knockdown (*n* = 6 each). ****: *p* < 0.01.

Given the observed abnormalities in cartilage formation across multiple skeletal elements following *hoxa4a* knockdown, we systematically investigated chondrocyte differentiation from cranial neural crest cells (CNCCs) in zebrafish embryos. Quantitative analysis revealed a marked reduction in CNCC proliferation at 24 h post-fertilization (hpf) in *hoxa4a* knockdown embryos compared to wild-type controls, as evidenced by phospho-histone H3 (PH3) immunostaining (*p* = 0.0006; Figure 5A,B). Applying TUNEL assays to quantify cell death, we observed that *hoxa4a*-deficient embryos contained substantially more apoptotic cells than their wild-type counterparts. The difference was highly significant (*p* < 0.0001), as shown in Figure 5C,D.

### 3.5. Effect of hoxa4a Knockdown on CNCCs and Pharyngeal Arch Primordia Formation

**Figure 6:**
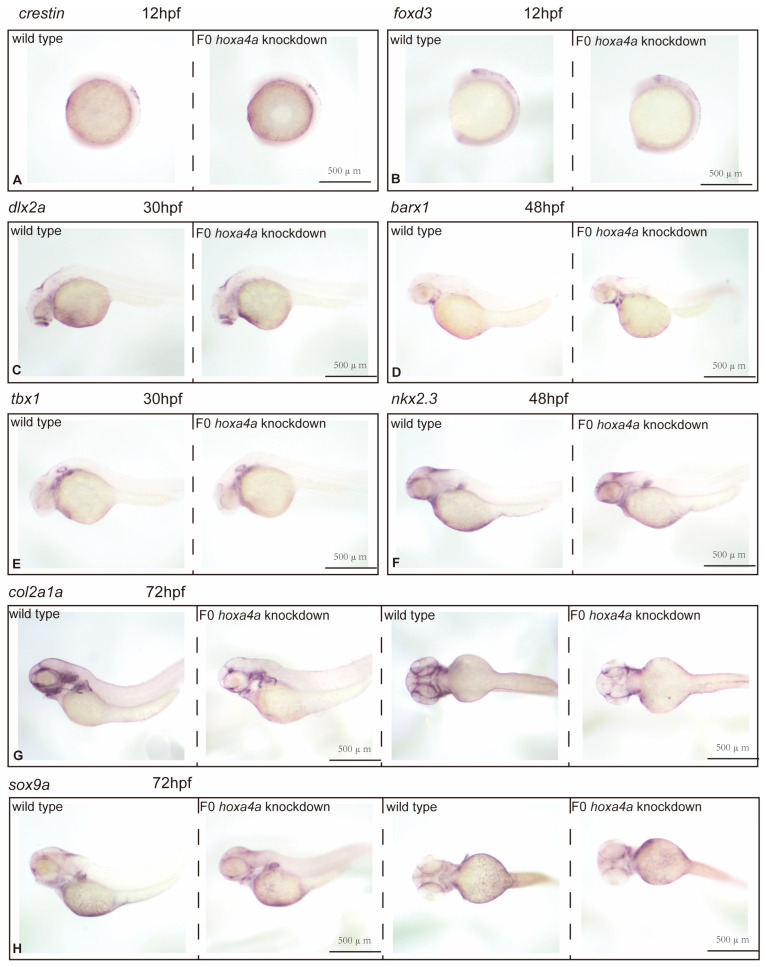
Expression of CNCC-associated markers during zebrafish development. ISH was performed to examine the following genes: (**A**) *crestin* and (**B**) *foxd3* at 12 hpf; (**C**) *dlx2a* at 30 hpf; (**D**) *barx1* at 48 hpf. No overt differences in the expression patterns of these four markers were detected between wild-type and *hoxa4a* knockdown embryos. (**E**) *tbx1* and (**F**) *nkx2.3* ISH shows comparable pharyngula formation in WT and *hoxa4a* knockdown embryos. (**G**,**H**) The expression of *col2a1a* and *sox9a* at 72 hpf was downregulated in *hoxa4a* knockdown than in wild-type embryos.

To assess the impact of hoxa4a knockdown on distinct developmental transitions from cranial neural crest cells (CNCCs) to chondrocytes, we examined marker genes associated with CNCC progression across multiple zebrafish embryonic stages using in situ hybridization. At 12 hpf, we measured *crestin* and *foxd3* transcripts (both expressed in migrating neural crest cells en route to the cranium) and found no difference between wild-type and F0 hoxa4a knockdown embryos. Thus, hoxa4a loss does not impair CNCC formation (Figure 6A,B). Quantitative analysis revealed comparable *dlx2a* expression patterns between wild-type and F0 *hoxa4a* knockdown embryos at 30 h post-fertilization (hpf), implying that *hoxa4a* does not play a critical role in the differentiation of CNCCs into pharyngeal arch-specific CNCC populations (Figure 6C). Similarly, spatial distribution analysis of *barx1* expression demonstrated no significant alterations in mesenchymal cell condensation between *hoxa4a* knockdown and control embryos at 48 hpf, further supporting the conclusion that *hoxa4a* is dispensable for this developmental process (Figure 6D).

### 3.6. Impact of hoxa4a Suppression on Pharyngula Development and Mesenchymal Cell Clustering

We performed ISH using *tbx1* and *nkx2.3*—both well-established pharyngula markers—to test the impact of *hoxa4a* suppression on pharyngula formation. No obvious differences in pharyngula segmentation or counts were detected between wild-type and F0 *hoxa4a* knockdown embryos. Thus, *hoxa4a* knockdown does not interfere with pharyngula development (Figure 6E,F).

### 3.7. Knockdown of hoxa4a Affects Pharyngeal Arch Cartilage Differentiation

We examined the chondrogenic markers *sox9a* and *col2a1a* at 72 hpf. Their expression in F0 *hoxa4a* knockdown embryos was significantly lower than in wild-type controls, showing a pronounced downregulation of both genes (Figure 6G,H). Together, these observations suggest that suppressing *hoxa4a* partially blocks the formation of differentiated chondrocytes and collagen.

## 4. Discussion

Distinguishing between environmental and genetic contributions to microtia etiology is challenging, particularly in sporadic cases lacking a clear family history. To overcome this research limitation, our previous investigation employed a unique study design involving six pairs of monozygotic twins exhibiting phenotypic discordance for microtia [8]. These twins were raised under identical environmental conditions and possessed nearly identical genetic profiles, allowing for the isolation of genetic factors contributing to microtia development. The study’s results indicated that the *HOXA4* variant (c.920A>C: p.H307P) likely represents a pathogenic mutation, as it was identified in multiple unrelated sporadic cases and was associated with both structural modifications and predicted functional impairment. This mutation results in histidine 307 mutating into proline, with the wild-type residue being positively charged and the mutant residue being non-polar, indicating that the mutant residue has stronger hydrophobicity than the wild-type residue. This amino acid residue is placed in the downstream region of the regulatory region, which is involved in protein transcriptional regulation and binds to DNA in a sequence-specific manner to form monomers or homo- and/or heterodimers. This variation may affect this process. Secondary structure prediction results indicate that mutations change the local secondary structure of the protein. Furthermore, analysis of the protein’s crystal structure revealed striking differences between the mutant and wild-type forms. These data suggest that this mutation is deleterious and may contribute to pathogenicity.

The etiology of microtia–atresia is considered to be heterogeneous, including both genetic and environmental factors that remain largely unknown. To date, three main hypotheses have been proposed to explain microtia–atresia and its associated craniofacial abnormalities: (1) Vascular disruption, which may hinder proper development of the first and second pharyngeal arches; (2) injury to Meckel’s cartilage, resulting in maxillofacial deformities; and (3) disruption of CNCC development, interfering with maxillofacial bone formation [29]. Cranial neural crest cells (CNCCs) derived from the neuroectoderm migrate into the pharyngeal arches, where they form the ectomesenchyme. During outer ear development, they migrate into the first and second pharyngeal arches, giving rise to connective tissue, pericytes, and vascular smooth muscle [30]. Before this, during gastrulation and neurulation, CNCCs undergo epithelial–mesenchymal transition (EMT) to detach from the neural plate and reach the pharyngeal arches [31]. In our study, PH3 and TUNEL assays revealed that hoxa4a knockdown significantly reduced CNCC numbers by suppressing proliferation and promoting apoptosis. Cheng et al. [32] noted that such a reduction can disrupt CNCC delamination and differentiation, ultimately causing chondrocyte disorganization and cartilage deformities. Therefore, the dysregulated apoptosis and proliferation of CNCCs in *hoxa4a* F0 mosaic knockdowns seem to play a part in craniofacial dysmorphologies.

CNCC condensation is a necessary precondition for chondrogenesis and plays a crucial role in determining the size and structure of the cartilage. During craniofacial development, cell migration and movements enable interactions between different cell and tissue types and their surroundings [33]. Following condensation, collagen II-encoding CNCCs express *col2a1a* and differentiate. Collagen II serves as the scaffold for the extracellular matrix (ECM) of cartilage and is essential for signal transduction between chondrocytes and the matrix, which maintains cartilage homeostasis. Skeletal dysmorphologies can result from *col2a1a* mutations, and reduced *col2a1a* expression in zebrafish induces craniofacial cartilage dysmorphologies [34,35]. *Sox9a* and *col2a1a* are typically expressed concurrently during chondrogenesis, with col2a1 functioning immediately downstream of *sox9a* [36]. SOX9 has two sequential critical roles in the development of the mandible; first in the specification and migration of CNCCs and second during chondrogenesis and formation of Meckel’s cartilage, the developmental precursor of the lower jaw [37]. Non-coding mutations at the far end of a large gene desert surrounding the SOX9 gene result in a human craniofacial disorder called Pierre Robin sequence. Previous studies have shown that neural crest specialization and migration occur normally in *sox9a* zebrafish mutants. But these mutants lack skull cartilage almost entirely and show decreased expression of *col2a1a*, with just a small number of hyoid bone cells remaining intact. According to the study’s findings, *sox9a* is not required for either pharyngeal arch cartilage aggregation or neural crest specialization [38]. Nevertheless, *sox9a* may regulate the later stage of CNCC-to-chondrocyte differentiation. Using in situ hybridization to monitor marker gene expression at successive steps of CNCC development, we found that both *sox9a* and *col2a1a* transcripts were reduced in *hoxa4a* knockdown zebrafish. Thus, the craniofacial cartilage defects observed upon *hoxa4a* loss likely stem from impaired CNCC development. It should be noted that our CRISPR/Cas9 strategy produced F0 mosaic knockdowns. While the observed craniofacial phenotypes were highly penetrant (>85% of injected embryos), the severity of cartilage defects could be somewhat variable due to the mosaic nature of mutations. Nevertheless, the consistent and striking mandibular hypoplasia across many embryos supports a key role of hoxa4a in pharyngeal arch development.

Hox genes are thought to confer positional identity along the embryonic anteroposterior axis. In higher vertebrates, they form four conserved clusters (HoxA–D), each containing up to 12 genes [39,40]. For instance, *Hoxa1* deletion leads to ectopic neurons that create an extra respiratory circuit at birth [41]. Hox genes act combinatorially and synergistically. A striking example is the mouse *Hox3* paralogs, expressed in pharyngeal pouch endoderm (*Hoxa3* only), hindbrain rhombomeres 5 and 6, and pharyngeal arches. While single knockouts of *Hoxa3* or *Hoxd3* show no obvious functional overlap, combined inactivation severely exacerbates the *Hoxa3*-null phenotype [42]. Hox4 paralogs, expressed in hindbrain r7 and r8, are thought to specify compartment identity. Earlier work showed coexpression of *hoxa4a*, *hoxb4a*, and *hoxd4a* in the posterior hindbrain, vagal ganglion, and branchial arches. Notably, at 1 dpf, all three hox4 paralogs are expressed in the pharyngeal arch mesoderm beneath and posterior to the otic vesicle [15]. This is consistent with the results we observed in our experiment. At 24 hpf, *hoxa4a* expression could be observed in the otic vesicle and dorsal hindbrain (Figure 2C,D). At 48 hpf, *hoxa4a* was detected in the pharyngeal arches, with this gene continuing to be expressed in the pharyngeal arches to 6 dpf (Figure 2E–L). According to their results, the Hox genes of group 4 regulate parts of the precerebellar and vagal systems as well as the development of pectoral fin neurons. Two or three genes may work in tandem or redundantly to carry out their respective functions. While our *hoxa4a* mosaic knockdown resulted in severe mandibular hypoplasia, the incomplete penetrance and the lack of obvious otic vesicle defects—despite *hoxa4a* expression in the otic vesicle at 24 hpf—may be partly explained by compensatory activity from *hoxb4a* and/or *hoxd4a*. Indeed, previous studies have shown that Hox4 paralogs can act redundantly in regulating pectoral fin neuron development and vagal system patterning. Whether these paralogs compensate for *hoxa4a* loss specifically in otic and certain pharyngeal arch contexts warrants further investigation using combinatorial knockdown or knockout approaches.

Several limitations of this study should be acknowledged. First, our CRISPR/Cas9 strategy produced F0 mosaic mutants rather than germline knockouts. Although the observed phenotypes were highly penetrant, mosaicism may contribute to variability in phenotypic severity and could obscure more subtle effects. Second, despite the substantial protein sequence conservation between human HOXA4 and zebrafish hoxa4a (54.7% identity), cis-regulatory elements controlling hoxa4a expression may have diverged significantly during evolution. Therefore, the spatiotemporal expression pattern of zebrafish hoxa4a might not fully recapitulate that of human HOXA4. Third, we did not perform rescue experiments by injecting wild-type or p.H307P mutant human HOXA4 mRNA into hoxa4a knockdown zebrafish. Such experiments would be essential to determine whether the human variant represents a hypomorphic allele, a dominant-negative mutation, or a gain-of-function change and to directly test its pathogenicity in vivo. Future studies using this approach are warranted to further clarify the molecular mechanism by which the HOXA4 p.H307P mutation contributes to microtia–atresia. Fourth, we did not conduct unbiased transcriptomic analyses (e.g., RNA seq) on pharyngeal arch-derived tissues from hoxa4a knockdown embryos. Genome-wide expression profiling could reveal additional downstream targets and pathways beyond sox9a and col2a1a that mediate hoxa4a function in craniofacial development. Addressing these limitations in future studies will provide a more complete understanding of the role of HOXA4 in microtia atresia.

## 5. Conclusions

**Figure 7:**
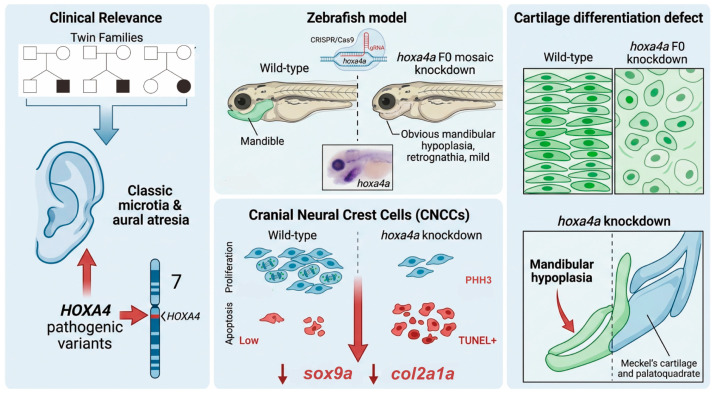
Schematic diagram of the *HOXA4* pathogenic mechanism.

In conclusion, this study identified *HOXA4* as a candidate gene implicated in the pathogenesis of microtia–atresia. Gene manipulation of *hoxa4a* in zebrafish using the CRISPR/Cas9 technique results in severe pharyngeal malformation and cartilage deformity. Knockdown of *hoxa4a* impaired cranial neural crest cell proliferation while promoting apoptosis, accompanied by significant downregulation of the chondrogenic markers *sox9a* and *col2a1a*. Consequently, pharyngeal arch chondrocytes displayed disorganized patterning and morphological defects, ultimately leading to mandibular hypoplasia (Figure 7). Together, these results demonstrate that *hoxa4a* plays a critical regulatory role in zebrafish craniofacial development.

## Data Availability

All figures are associated with raw data. Raw images can be provided upon request. Sequences used for CRISPR/Cas9 and in situ hybridization are included in the Supplementary Materials.

## Supplementary Materials

The following supporting information can be downloaded at: https://www.mdpi.com/article/10.3390/jdb14020022/s1. Table S1. Sequences used in Cas9/gRNA design and knockout validation PCR. Table S2. Sequences of probes used in in situ hybridization.

## References

[1] Zou Y. (2023). Congenital ear malformation (CEM). Acta Otolaryngol..

[2] Bartel-Friedrich S. (2015). Congenital Auricular Malformations: Description of Anomalies and Syndromes. Facial Plast. Surg..

[3] King J.A., Marker P.C., Seung K.J., Kingsley D.M. (1994). BMP5 and the molecular, skeletal, and soft-tissue alterations in short ear mice. Dev. Biol..

[4] Shih H.Y., Hsu S.Y., Ouyang P., Lin S.J., Chou T.Y., Chiang M.C., Cheng Y.C. (2017). Bmp5 Regulates Neural Crest Cell Survival and Proliferation via Two Different Signaling Pathways. Stem Cells.

[5] Guajardo H.M., Hatini P.G., Commons K.G. (2021). The mouse dorsal raphe nucleus as understood by temporal Fgf8 lineage analysis. J. Comp. Neurol..

[6] Zhang C., Zhang J., Wang J., Yan Y., Zhang C. (2020). Alpha-fetoprotein accelerates the progression of hepatocellular carcinoma by promoting Bcl-2 gene expression through an RA-RAR signalling pathway. J. Cell. Mol. Med..

[7] Cox T.C., Camci E.D., Vora S., Luquetti D.V., Turner E.E. (2014). The genetics of auricular development and malformation: New findings in model systems driving future directions for microtia research. Eur. J. Med. Genet..

[8] Fan X., Ping L., Sun H., Chen Y., Wang P., Liu T., Jiang R., Zhang X., Chen X. (2020). Whole-Exome Sequencing of Discordant Monozygotic Twin Families for Identification of Candidate Genes for Microtia-Atresia. Front. Genet..

[9] Jain K., Sykes V., Kordula T., Lanning D. (2008). Homeobox genes Hoxd3 and Hoxd8 are differentially expressed in fetal mouse excisional wounds. J. Surg. Res..

[10] Cheng S., Qian F., Huang Q., Wei L., Fu Y., Du Y. (2018). HOXA4, down-regulated in lung cancer, inhibits the growth, motility and invasion of lung cancer cells. Cell Death Dis..

[11] Holve S., Friedman B., Hoyme H.E., Tarby T.J., Johnstone S.J., Erickson R.P., Clericuzio C.L., Cunniff C. (2003). Athabascan brainstem dysgenesis syndrome. Am. J. Med. Genet. A.

[12] Chen T., Li Q., Xu J., Ding K., Wang Y., Wang W., Li S., Shen Y. (2007). Mutation screening of BMP4, BMP7, HOXA4 and HOXB6 genes in Chinese patients with hypospadias. Eur. J. Hum. Genet..

[13] Bhatlekar S., Addya S., Salunek M., Orr C.R., Surrey S., McKenzie S., Fields J.Z., Boman B.M. (2014). Identification of a developmental gene expression signature, including HOX genes, for the normal human colonic crypt stem cell niche: Overexpression of the signature parallels stem cell overpopulation during colon tumorigenesis. Stem Cells Dev..

[14] Yamashita T., Tazawa S., Yawei Z., Katayama H., Kato Y., Nishiwaki K., Yokohama Y., Ishikawa M. (2006). Suppression of invasive characteristics by antisense introduction of overexpressed HOX genes in ovarian cancer cells. Int. J. Oncol..

[15] Punnamoottil B., Kikuta H., Pezeron G., Erceg J., Becker T.S., Rinkwitz S. (2008). Enhancer detection in zebrafish permits the identification of neuronal subtypes that express Hox4 paralogs. Dev. Dyn..

[16] Alasti F., Sadeghi A., Sanati M.H., Farhadi M., Stollar E., Somers T., Van Camp G. (2008). A mutation in HOXA2 is responsible for autosomal-recessive microtia in an Iranian family. Am. J. Hum. Genet..

[17] Brown K.K., Viana L.M., Helwig C.C., Artunduaga M.A., Quintanilla-Dieck L., Jarrin P., Osorno G., McDonough B., DePalma S.R., Eavey R.D. (2013). HOXA2 haploinsufficiency in dominant bilateral microtia and hearing loss. Hum. Mutat..

[18] Kessler S., Minoux M., Joshi O., Ben Zouari Y., Ducret S., Ross F., Vilain N., Salvi A., Wolff J., Kohler H. (2023). A multiple super-enhancer region establishes inter-TAD interactions and controls Hoxa function in cranial neural crest. Nat. Commun..

[19] Kimmel C.B., Ballard W.W., Kimmel S.R., Ullmann B., Schilling T.F. (1995). Stages of embryonic development of the zebrafish. Dev. Dyn..

[20] Liu C., Li R., Li Y., Lin X., Zhao K., Liu Q., Wang S., Yang X., Shi X., Ma Y. (2022). Spatiotemporal mapping of gene expression landscapes and developmental trajectories during zebrafish embryogenesis. Dev. Cell.

[21] SWISS-MODEL. https://swissmodel.expasy.org.

[22] PROVEAN. http://provean.jcvi.org/index.php.

[23] Polymorphism Phenotyping v2. http://genetics.bwh.harvard.edu/pph2/.

[24] Wu R.S., Lam I.I., Clay H., Duong D.N., Deo R.C., Coughlin S.R. (2018). A Rapid Method for Directed Gene Knockout for Screening in G0 Zebrafish. Dev. Cell.

[25] Montague T.G., Cruz J.M., Gagnon J.A., Church G.M., Valen E. (2014). CHOPCHOP: A CRISPR/Cas9 and TALEN web tool for genome editing. Nucleic Acids Res..

[26] Chang N., Sun C., Gao L., Zhu D., Xu X., Zhu X., Xiong J.W., Xi J.J. (2013). Genome editing with RNA-guided Cas9 nuclease in zebrafish embryos. Cell Res..

[27] Wang Y., Ping L., Luan X., Chen Y., Fan X., Li L., Liu Y., Wang P., Zhang S., Zhang B. (2020). A Mutation in VWA1, Encoding von Willebrand Factor A Domain-Containing Protein 1, Is Associated With Hemifacial Microsomia. Front. Cell Dev. Biol..

[28] ZESTA Database. https://db.cngb.org/stomics/zesta/.

[29] Chen Q., Zhao Y., Shen G., Dai J. (2018). Etiology and Pathogenesis of Hemifacial Microsomia. J. Dent. Res..

[30] Schilling T.F., Kimmel C.B. (1994). Segment and cell type lineage restrictions during pharyngeal arch development in the zebrafish embryo. Development.

[31] Soldatov R., Kaucka M., Kastriti M.E., Petersen J., Chontorotzea T., Englmaier L., Akkuratova N., Yang Y., Haring M., Dyachuk V. (2019). Spatiotemporal structure of cell fate decisions in murine neural crest. Science.

[32] Cheng X., Li H., Yan Y., Wang G., Berman Z., Chuai M., Yang X. (2017). From the Cover: Usage of Dexamethasone Increases the Risk of Cranial Neural Crest Dysplasia in the Chick Embryo. Toxicol. Sci..

[33] Murillo-Rincon A.P., Kaucka M. (2020). Insights Into the Complexity of Craniofacial Development From a Cellular Perspective. Front. Cell Dev. Biol..

[34] Williams A.L., Bohnsack B.L. (2022). Zebrafish Model of Stickler Syndrome Suggests a Role for Col2a1a in the Neural Crest during Early Eye Development. J. Dev. Biol..

[35] Zhang T., Sun X., Li M., Huang H. (2021). De novo mutation in COL2A1 leads to lethal foetal skeletal dysplasia. Bone.

[36] Zhao Q., Eberspaecher H., Lefebvre V., De Crombrugghe B. (1997). Parallel expression of Sox9 and Col2a1 in cells undergoing chondrogenesis. Dev. Dyn..

[37] Long H.K., Osterwalder M., Welsh I.C., Hansen K., Davies J.O.J., Liu Y.E., Koska M., Adams A.T., Aho R., Arora N. (2020). Loss of Extreme Long-Range Enhancers in Human Neural Crest Drives a Craniofacial Disorder. Cell Stem Cell.

[38] Yan Y.L., Miller C.T., Nissen R.M., Singer A., Liu D., Kirn A., Draper B., Willoughby J., Morcos P.A., Amsterdam A. (2002). A zebrafish sox9 gene required for cartilage morphogenesis. Development.

[39] Akam M. (1989). Hox and HOM: Homologous gene clusters in insects and vertebrates. Cell.

[40] Duboule D. (2007). The rise and fall of Hox gene clusters. Development.

[41] del Toro E.D., Borday V., Davenne M., Neun R., Rijli F.M., Champagnat J. (2001). Generation of a novel functional neuronal circuit in Hoxa1 mutant mice. J. Neurosci..

[42] Manley N.R., Capecchi M.R. (1998). Hox group 3 paralogs regulate the development and migration of the thymus, thyroid, and parathyroid glands. Dev. Biol..

