# Supplementary material for: Functional and Genetic Analyses Unveil the Implication of hoxa4a in Zebrafish Craniofacial Development
Source: J Dev Biol. 2026 May 15;14(2):22. doi: 10.3390/jdb14020022 (PMC13214747; doi:10.3390/jdb14020022)
Supplement: Supplementary file 1 [file jdb-14-00022-s001.zip › jdb-4209855-supplementary.pdf]

Table S1. Sequences used in Cas9/gRNA design and knockout validation PCR

Function	Type	Sequence (5'-3')
Cas9/gRNA design*	T7-promoter	TAATACGACTCACTATA
	gRNA target R1	GGTGATATTCCTCGCAGGGT
	gRNA target R2	GGGTCCATGATTCTGCGAAA
	gRNA target R3	GCTGACTCGACGTCGACGTG
	gRNA target R4	GACTATAGGTTAGAGGAACA
	Scaffold sequence	GTTTTAGAACTAGAAATAGC
	Tracr rev sequence	AAAAAAAGCACCGACTCGGTGCCAC
Knockout validation PCR	R1-Forward	CATTGGTGCTTGTTTACACGAT
	R1-Reverse	GCTACAGTCCTTGTTTCCATCC
	R2-Forward	TGGGTTTTGGAGAGATCAGAAT
	R2-Reverse	ACAACTGGGTTTCCTACATGCT
	R3-Forward	TTAAAGATCGTGGAAGAGGGAA
	R3-Reverse	AAGTAACCCTGTGAAGTTCCGA
	R4-Forward	AGGCTCTTGAGCTTGAAAAAGA
	R4-Reverse	CACTATAGCGTTGATTCCCACA

* The structure of the forward primer is T7-promoter – R1/R2/R3/R4 – Scaffold sequence.

Table S2 . Sequences of probes used in *in situ* hybridization

Gene	Primer type	Sequence (5'-3')
crestin	Forward	CAGAAGCCCTCATCAGAGAGTTG
	Reverse	GTTGCTTGTCAGGCAGAATCAGG
foxd3	Forward	CCTACTCGTACATCGCCCTC
	Reverse	CCGGGTTAAGGACAGGGAC
dlx2a	Forward	CACAGTTCTGCTTTGCGTCG
	Reverse	CCCAAGTCGGCAGAGTCAAA
barx1	Forward	CTGGGCGGATCAGACTTCTC
	Reverse	GCTTCTCGTGTCCTCTCCTG
tbx1	Forward	GCAGCTGTCCCATTTTTGCG
	Reverse	ACGGCGGTAAATCTGGTCTC
nkx2.3	Forward	TCGTGTTTTCTCGGAGGTGG
	Reverse	GCGCATTAGTGGACGTGTTC
sox9a	Forward	CCTCGACCCCTACCTGAAGA
	Reverse	GGCGGGAGGTATTGGTCAAA
col2a1a	Forward	TCTGAAGTCCATCAACGGGC
	Reverse	TTTTCCGTCACGCTAAACGC
